# Recent Advances in Techniques for Starch Esters and the Applications: A Review

**DOI:** 10.3390/foods5030050

**Published:** 2016-07-09

**Authors:** Jing Hong, Xin-An Zeng, Charles S. Brennan, Margaret Brennan, Zhong Han

**Affiliations:** 1 School of Food Science and Engineering, South China University of Technology, Guangzhou 510641, Guangdong, China; hongjingjudy@126.com (J.H.); fezhonghan@scut.edu.cn (Z.H.); 2 Centre for Food Research and Innovation, Department of Wine, Food and Molecular Biosciences, Lincoln University, Lincoln 85084, New Zealand; charles.brennan@lincoln.ac.nz (C.S.B.); margaret.brennan@lincoln.ac.nz (M.B.)

**Keywords:** starch, esterification, dual modification, activation, catalyst, microwave, pulsed electric fields, high pressure, high temperature, oxidation, cross-linking, application

## Abstract

Esterification is one of the most important methods to alter the structure of starch granules and improve its applications. Conventionally, starch esters are prepared by conventional or dual modification techniques, which have the disadvantages of being expensive, have regent overdoses, and are time-consuming. In addition, the degree of substitution (DS) is often considered as the primary factor in view of its contribution to estimate substituted groups of starch esters. In order to improve the detection accuracy and production efficiency, different detection techniques, including titration, nuclear magnetic resonance (NMR), Fourier transform infrared spectroscopy (FT-IR), thermal gravimetric analysis/infrared spectroscopy (TGA/IR) and headspace gas chromatography (HS-GC), have been developed for DS. This paper gives a comprehensive overview on the recent advances in DS analysis and starch esterification techniques. Additionally, the advantages, limitations, some perspectives on future trends of these techniques and the applications of their derivatives in the food industry are also presented.

## 1. Introduction

**Figure 1:**
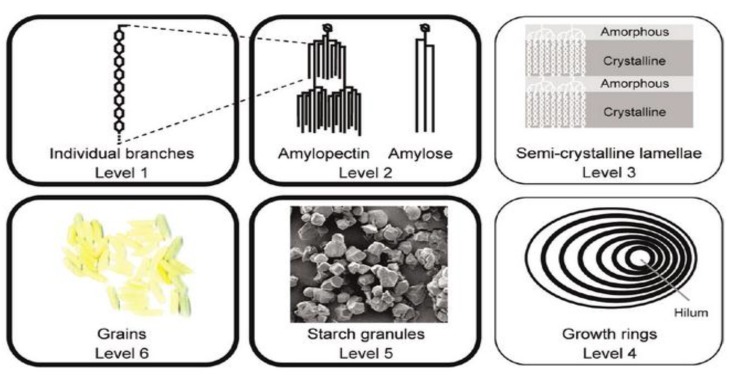
Six levels of starch granule structure: level 1of individual branches, level 2 of amylose and amylopectin, level 3 of semi-crystalline lamellae, level 4 of growth rings, level 5 of starch granules, level 6 of cereal grains [2].

Starch is an inexpensive, abundantly available, widely used, and naturally produced polysaccharide molecule found in fruits, seeds, stems, tubers and roots for the storage of solar energy [1]. It exists in six structural levels (seen in Figure 1), grains; granules; growth rings; semi-crystal layers, which are between the crystalline and amorphous area; molecules involving linear and branched molecules linked by α-(1 → 6) bonds in branching points forming amylopectin; and individual linear branch linked by α-(1 → 4) linear links forming amylose [2,3,4]. The different quantity and organizational distribution of amylose and amylopectin leads to diverse starch compositions, affecting their structures and functions. Due to the diversity in structure and function, such as water-solubility, instability of paste under acid conditions, heating and shearing reactions, native starches generally pose problems in industrial applications. To satisfy the requirement of consistency, and to expand desirable functional properties, free available hydrophilic hydroxyl groups of starch are replaced by hydrophobic substitutions through glycosidic bond cleavage [5,6,7]. This process of modifying starch has been used in a range of industrial areas, particularly in food production due to its good filming properties and excellent emulsion-stabilizing properties [8,9]. Table 1 summarizes the relevant esterification studies of different type starches by conventional modification and dual modification involving physical, chemical and enzymatic techniques to provide modified starches of premium quality.

These dual modification techniques, based on conventional methods, have been optimized by the use of different cultivars [10], pH [11], fatty acids [12], anhydride type [13], temperature [14], reagent catalyst [11], enzyme catalyst [15], dual enzyme modification [16], high pressure [14] and microwave technology [17].

**Figure 2:**
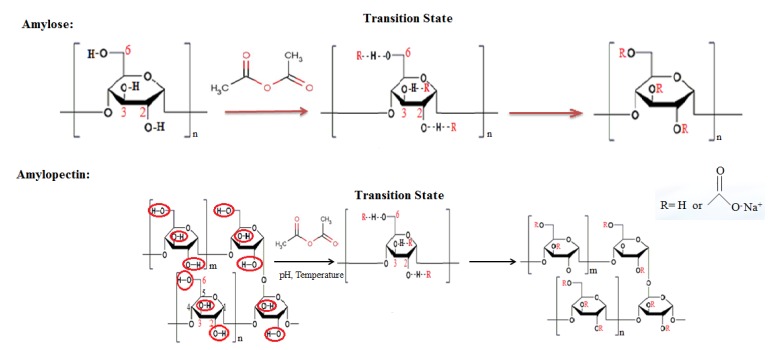
The reaction mechanism of starch esterification (amylose and amylopectin). This scheme took acetic anhydride as an example.

The reaction mechanism of esterification is shown in Figure 2. The covalently linked components generally react with any available hydroxyl moiety of accessible glucose monomers to impart desirable qualities to the starch substrate. For example, in acetylated starch, the hydroxyl groups of starch are substituted by acyl groups resulting in an amphiphilic character, which weakens the binding force among the starch molecules [18,19]. The new starch esters are characterized by their degree of substitution (DS) as an indicator of their quality and physical properties [20]. Zieba et al. [21] did research on starch acetates with an equal DS of ~0.1 and found that there was a positive correlation between resistance to amylolysis and the number of acetyl groups substituted at carbon atoms 2 and 3. The DS of a starch ester has a role in the extent to which a starch is able to recrystallize or retrograde. These ingredients have been successfully utilized in the food industry for more than half a century, as emulsifiers encapsulating material, films and coatings, and in the manufacturing of gel type products [22,23].

There have been few publications focusing on the manufacture and application of starch esters within the food industry. Both Zukowska et al. [24] and Abbas et al. [25] have illustrated the use of modified starches in the commercialization of food products, whilst Alissandratos et al. [26] studied the applications in pharmaceutical products. Zuo et al. [27] optimized a different dry method to study the effect of maleic anhydride on esterifying corn starch granules. Golachowski et al. [28] reviewed the alternated properties of starch esters, especially for acetylated starches. More recently, Sweedman et al. [29] discussed the use of structural modifications to starch granules in terms of their physico-chemical properties, focusing on the potential economic and environmental aspects of this process. Much of the interest in modified starches has been concentrated on dual modification processes and their effects on DS value, except for the study of Ashogbon et al. [1], who overviewed the similarities and differences among starch esters from different botanical sources.

This review, attempts to redress the balance, summarizes recent advances in esterification procedures, including conventional and dual modification, such as catalyst addition, ball milling activation, high temperature/pressure, microwave assist, pulsed electric fields, chemical synthesis involving cross-linking, and oxidation.

## 2. Determination Methods of DS

A commonly used parameter when considering the quality and performance of starch esters is the DS, which relates to the average number of hydroxyl groups replaced by acetyl groups or other derivatives per glucose unit and serves as an indicator of the its properties and applications [18,38]. The DS can be measured by several techniques, including titration, nuclear magnetic resonance (NMR), Fourier transform infrared spectroscopy (FT-IR) and headspace gas chromatography (HS-GC). The assets and drawbacks of the determination methods are shown in Table 2.

Titration and back-titration based on color change of the indicator are both widely accepted and used in industrial, as well as laboratory, settings [13,39,40]. Miao et al. [39] dispersed octenyl succinic anhydride modified starch into a hydrochloric acid/isopropanol solution in order to eliminate Cl– and diminish error. NaOH solution was utilized to neutralize and titrate carbonyl groups by using an end-point indicator, however, this method can be affected by the CO_2_ that is present in the atmosphere, as it can dissolve into the solution causing non-avoidable experimental error. Back-titration, however, relies on the saponification of the product using NaOH or KOH to hydrolyze the acetyl groups followed by titration of the excess alkali which thus avoids disturbing of CO_2_ in the atmosphere therefore removing this source of error [13], however, being dependent on titration, both of these methods are time consuming and liable to human error.

NMR and FT-IR are systematic methods that have been used to measure the DS of starch esters. ^1^H-NMR and ^13^C-NMR may be employed for evaluating succinate, alkenylsuccinate and some acetate esters by adding sodium 3-(trimethylsilyl) propionate 2,2,3,3-D4 as an internal standard [32,41]. FT-IR can be used to characterize starch acetate standards by integrating titration methods [14]. Researchers have measured the diverse DS of starch esters by titration and further characterization by FT-IR, where their standard curves A1743 of C=O vibration (1743 cm^−1^) related to A2929 of starch CH2 vibration (2929 cm^−1^) were regressed to predicted other samples. 1H-NMR and FT-IR analyses require complicated procedures however, more and more researchers employ this method to determine the precise DS value [42,43,44]. Thermal gravimetric analysis/Infrared spectroscopy (TGA/IR) can also be used to determine DS, which need the sample quality of 8 to 11 mg. The analysis of the DS mainly depended on a set of standard samples and these standards were analyzed by the hydrolysis method first. The reference has confirmed that it was possible to predict the DS by TGA using derivatives of thermograms (DTG), however, this method still needs the detection of DS by titration which was time-consuming [45].

Another method that has been employed to estimate DS of anhydride modified cellulose is the combination of headspace and gas chromatography (HS-GC) proposed by Zhong et al. [46]. The research outlined how the DS values were measured by detecting GC signal of CO_2_, which was produced by neutralization of carboxyl groups and the added bicarbonate solution in sealed vials. Therefore, HS-GC is another simple and automated method for determining DS of anhydride-modified cellulose, which can be used in assessing the DS of starch esters.

## 3. Comparison of Conventional and Dual Modification Methods

### 3.1. Conventional Modification

Starch esters modified by conventional method have already been widely applied in industry by converting the hydroxyl groups of starch into diverse derivatives. There is a mass of factors affecting the end products with different DS, such as starch cultivar, reactant concentration (starch slurry, acid/anhydride reactant), acid or alkali, reaction time, temperature, pH moderation, reaction medium and the presence or absence of a catalyst, which is illustrated in Table 1.

Different starch cultivars exhibit different amylase/amylopectin ratios. As early as 1996, Fringant et al. [53] studied the behavior of starches with high amylose content and high amylopectin content when treated by acetylation. They deduced that high amylopectin acetates in starch-based materials can influence its physicochemical properties, and they become rigid and brittle because of the strong interaction and low entanglement among molecules. Liu et al. [54] studied the differences between normal and waxy rice starch and found that waxy modified starch was freeze thaw stable compared to the freeze behavior of normal starch, as identified by Agboola [55]. Their work compared modified A-type starch and B-type starch under similar processing conditions, both of these modified starches appeared to exhibit reduced gelatinization temperature, enthalpy (ΔH), and higher solubility, swelling power, and better paste clarity when compared with native starch. Modified B-type had greater DS, larger but more fragile granules with different granule size, and crystal forms [15,30,34]. Miao et al. [39] selected soluble starch of sugary maize as the raw material, which was modified by octenyl succinic anhydride. Their study revealed that the modified soluble starch could create a stable oil-in-water emulsion. Similarly, other researchers have investigated the hydrolysis of potato starch after being treated by acetic anhydride [33]. Results confirm that the modified starch had an effect on the susceptibility to enzymatic hydrolysis, especially in the membrane reactor, which meant that the higher the DS was, the greater the reduction in the susceptibility to amylolysis and the hydrolysate of acetylated starch in the surface/interfacial tension was. This would be interesting to explore in the regulation of starch digestion as a potential cure of vascular diseases and for bio-pharmaceuticals industries.

In recent years, in order to develop more applications of esterified starch, researchers have focused on health care applications, as well as new composite materials for nanocomposite film development [39,56]. Foods (bread, steamed-bread, noodles, rice and fast foods) that we consume regularly are produced by normal native starch and generally tend to have a high glycemic index during digestion [57]. Han et al. [58] and Miao et al. [39] conducted research on yielding resistant starch through esterification. Their investigations revealed that more slowly digesting starch and resistant starch were produced after esterification. These findings have the potential to develop a novel approach to create nutritional foods and a balanced diet. This cannot only help to fight against the epidemics of diabetes and obesity through low glycemic response foods, but also improve the nutritional quality of foods. For instance, Katerinopoulou et al. [58] developed a process of making new composite materials of nanocomposite film based on acetylated starch.

Research has used these starch esters with lower DS of 0.01–0.20 in applications for food formulation and other areas, such as film forming, binding, adhesion, thickening, stabilizing and texturing [10,50,59]. Starch esters with higher DS are now used for their properties of solubility, thermoplasticity, hydrophobicity and other values in non-food application, such as tablet binders, hot melt adhesives, coating, cigarette filters, biodegradable packaging materials and pharmaceutical aspects [14,60].

### 3.2. Dual Modification Combined with Physical Methods

Dual modification is the combination of conventional modification and other kinds of techniques to improve the properties of starch esters. These dual modification methods include chemical reaction in the presence of a specific physical environment or an enzymatic treatment to increase the yield of derivatizations or improve the DS [8]. The common dual methods with physical motivation are catalyst-added, ball milling before esterification, synthesis of high temperature, high pressure, microwave, and pulsed electric fields.

#### 3.2.1. Catalyst

A catalyst is an active factor that accelerates reaction rates and shortens reaction times. According to the origin of the catalyst, it can be divided into two types involving chemical reagents or enzymatic materials.

Acetylated starch with a low DS of 0.01–0.20 is usually studied in an aqueous medium in the presence of alkaline catalyst. In recent years, the introduction of a catalyst-used reaction was proposed to increase DS by Wang et al. [11]. Sodium hydroxide and other alkali metals and alkaline-earth metal hydroxides were used as the catalyst in this study. Wang et al. [61] then reported a more detailed synthesis procedure in a non-aqueous medium of pyridine and manufactured the esterified starch with a quite high DS (>3), which was then used as a delivery carrier for bioactive food components. Notwithstanding the positive effects on the reaction efficiency and high DS, this method has not been applied to mass production because of the toxicity of pyridine. In addition, Fei et al. [62] pointed out that using *p*-toluenesulfonic acid as a catalyst to produce starch esters had a great effect on the DS value, which played an essential role on increasing the collision times of molecules among starch and acetic anhydride, resulting in higher DS.

Use of an enzyme as a catalyst is an alternative way to accelerate reaction rates and improve reaction efficiencies. Singh et al. [17] considered a biocatalyst-assisted reaction as an environmentally friendly method under milder conditions, and this was found to be a useful procedure for starch esterification. Taking a wider view of the research, lipase enzymes can be a generally be adopted as catalyst agents. Rajan et al. [37] discussed the effect of a lipase catalyst to manufacture a higher level of DS (1.10) with the assistance of microwaves, and Huang et al. [63] found that pretreatment of oat starch with α-amylase gave a lower DS. This method is a feasible technique to increase the starch granule surface area, thus making it easier for the chemical reagent to infiltrate the inner parts of granules, resulting in the distinct alteration on the retrogradation properties, freeze-thaw stability and resistant stability. It is possible, therefore, to use multistep enzyme procedures to produce well-characterized DS by manipulating the enzyme activity of amylases, lipases and even proteases. All of these enzymes will play a vital role in creating new starch structures, and, hence, starches with unique physico-chemical properties.

To sum up, even though the use of an organic solvent as catalyst can improve the reaction efficiency, the challenges of solvents’ poor environmental image and high cost make it difficult to expand extensive application into industry. By contrast, the enzyme catalyst-assisted methods are environmentally friendly with few by-products and can therefore be used directly.

#### 3.2.2. Ball Milling Activation

Ball milling, as a lower-cost and physically-modified method, has been used to enlarge the surface area of molecules. Friction, collision, impingement, shear or other mechanical actions are induced during ball milling [64], these mechanical actions can increase the reaction surface of starch molecules and, consequently, are responsible for an increase in the reaction efficiency. Tian et al. [65] took cassava starch of C-type crystal structure and found that micronized or mechanical activation was a profitable means by which to improve DS and was suitable for use in the encapsulation of sensitive food ingredients. When the starch cultivar was replaced by rice starch of an A-type crystal structure, it was noted that the milling time was prolonged, mechanical activation gradually accelerated the reaction efficiency (from 53% to 88%), which was also accompanied by an increased DS (from 0.013 to 0.02) [66]. It is worth mentioning that the DS value and reaction efficiency are not able to be progressively increased after the milling time reaches 50 h, and this still requires further research on the reaction mechanics involved. For the most part, pre-mechanically activated starch can reduce the use of chemical reagents and the derivatives can be safely used as substitutes for encapsulation of sensitive food ingredients.

#### 3.2.3. High Temperature/Pressure

Other effective, physical methods to active starch granules utilize harsh circumstances, such as high temperature or high pressure. Shogren [67] discovered an effective step to accelerate reaction by heating the starch granules to 120–128 °C; they concluded that starch acetates produced in this way were important in foam packaging for food and other potential applications, such as biodegradable soil drainage aids, seed propagation blocks, and bio-friendly replacements for polystyrene foam and vermiculite/perlite. Similarly, Shogren [14] used a replica reaction temperature of 120 °C to dry corn starch in a vacuum oven, while a higher temperature of 180 °C was continuously applied to achieve esterified starch. The improved procedure greatly promoted DS in only 20 min, which, as expected, reduced the reaction time drastically [68]. Vaca-Garcia [69] has since used a much higher reactive temperature of 180–230 °C to produce esterified starch, however, this method is under patent so no other details are available. Compared to the conventional method in aqueous medium, the improved technique proved to be simpler and more economical after an extra step of high temperature/pressure, taking place before or as part of the reaction process to maximize reaction efficiency and reduce reaction time.

#### 3.2.4. Microwave Technology

Microwaves are known to be non-ionizing energy, which can generate heat in the penetrated medium by the “molecular friction” in an alternating electromagnetic field. When microwave technology was used in esterification, Jyothi et al. [36] combined microwave radiation with heating of 140 °C, which produced the succinate derivatived starch with DS of 0.007–0.051. Even though the research had little improvement on DS, it provided other researchers with a novel method to yield starch esters and promoted the development of starch esterification. Subsequently, Biswas et al. [51] improved the method to obtain a much higher DS of 0.30 using microwave-assisted modification within only 5 min. Singh et al. [17] continued to study microwave-assisted esterification of soluble starch, applying the innovative technique of iodomethane and 30% potassium hydroxide accompanied by microwave irradiation, soluble starch can yield a reaction efficiency of 72% within the much shorter time of 4.66 min. This same approach was also used by Rivero et al. [70] to obtain octenyl succinic anhydride starch, which was employed as compatibilizer for linear low density polyethylene/starch blends. In comparison to these starch esters modified by the conventional and microwave-assisted esterification, the latter had better application properties as a compatibilizer for the blends. Rajan et al. [15] proposed that the microwave-assisted transformation of the starches did not affect the distribution of molecular weight and granular structure but provided more energy to break the covalent bonds in glucose units, as compared to when these samples were treated by conventional methods [31,71]. Makuuchi [72] proved that employing microwave radiation at higher powers may have some potential to degrade starch. When using 5–20 min of microwave to prepare methylated starch, it was noted that the DS of methyl starches increased and then decreased along with the increase of microwave time, and explored the highest DS with a microwave time of 12 min, as studied by Hou et al. [73].

More researchers and entrepreneurs are choosing to employ ecologically friendly procedures that use less volatile organic solvents, avoid toxic solvent consumption, and provide better economic benefits. In view of the above research, due to its energy saving, high conversion rate, speed of reaction and existing applications, microwave-assisted esterification has many advantages that make it a popular, emerging and promising technique.

#### 3.2.5. Pulsed Electric Fields

Pulsed electric fields (PEF), as a promising non-thermal technique, has been widely used for inactivation of microorganisms and enzymes in food applications. In addition to this, however, researchers have reported the effects of PEF treatment on starch modification with different types, including A-type (corn starch), B-type (potato starch) and C-type (cassava starch) [3,4]. These researchers focused on the physicochemical properties, pasting properties, thermal stability, structure, and granule morphology under a series of electric field strengths, and proposed that the decreased relative crystallinity is a result of damage to the surface of the granules. However, there are few studies concerned with the synergy of esterification and PEF [35,74]. Recently, those authors studied the effect of PEF-assisted acetylation on morphological, structural and functional characteristics of potato starch (B-type) and cassava starch (C-type). Results showed that PEF treatment can be a potentially beneficial method for acetylation, and achieves higher DS with a shorter reaction time. It also exhibited good freeze-thaw stability and, therefore, has potential applications in the frozen food industry.

Thus, PEF, combined with esterification, could be a novel and promising technique to improve reaction conditions and efficiency by modifying its properties. There are still many challenges for PEF–assisted esterification, including understanding the transformation of the fractal nanostructure, however, there are also opportunities for more applications in new areas.

### 3.3. Dual Modification Combined with Chemical Methods

The combination of esterification with other chemical modification methods, such as cross-linking and oxidation, have already been studied. These starch esters are prepared by synthetic methods and, due to double functional groups being present in the starch molecules, dual properties are exhibited.

#### 3.3.1. Cross-Linking

Simple modification of cross-linking, where the polymer chains of starch cross-link with two- or poly-function compounds, such as adipic acid, citric acid or phosphoric acid, leading to a significant increase in molecular weight, is a well established and widely used chemical method for starch modification in the laboratory, as well as in industry [75].

The integration of esterification and cross-linking is an effective way to obtain the derivatives with double functions. Han et al. [58] explored cross-linked acetylated starch, focusing on its slowly digestible starch content. They found that a higher content of slowly digestible starch was present when dual modification was employed as opposed to those modified by cross-linking or acetylation alone. Slowly digestible starch is beneficial to diabetics and those with cardiovascular disease, as has been proved by subsequent research [16]. Das et al. [76] conducted further study on the characteristics of dual modified starch esters, and pointed out that the esterified crosslinked starch, not only had good emulsification stability, but also showed high resistance to heat, acid, and shear force with DS of 0.018–0.058. Lopez et al. [77] carried out a systematic analysis of the physicochemical and functional properties of corn starch using the combined techniques. The study found that the acetyl content of acetylated-crosslinking starch (1.9%) was slightly lower than that of single acetylated starch (2.2%), which indicated that the hydroxyl groups had been replaced by an amount of cross-linking, leading to fewer locations being available and, thus, a greater resistance to importing acyl groups. Hence, this method of cross-linking and esterification can obtain more different functional properties and enhance the application in food products.

Based on the mentioned studies, therefore, it appears that it is still necessary to take up the challenge to establish novel methods in order to produce starch esters with double functional groups with a high rate of efficiency, so as to cater to a more universal applications in different areas.

#### 3.3.2. Oxidation

Oxidation as a form of chemical modification, involves the introduction of carboxyl and carbonyl functional groups by means of sequential depolymerization of the starch. Such starches have been established to be whiter in color, but have low values in breakdown and setback viscosity when pasting properties are analyzed [78]. There are a few researchers studying starch modified by the combination of esterification and oxidization. Aini et al. [79] studied the esterification of starch followed by oxidization, and concluded that the gelatinization temperature, peak viscosity and retrogradation were lower than starch that had only been subjected to esterification. Additionally, the absorption of metal ions can occur in the oxidized starch and is common in acetylated crosslinked starch [80]. This diversifies the application of oxidized esterified starch into the area of adsorption of metal ions. It is apparent then that starch esters, assisted by chemical synthesis, can drive the double functions and be applied in non-food industries.

## 4. Emerging Processing Trends in the Future

The preparation of starch esters is a well-established field of research. The microwave-assisted method mentioned above is very convenient and eco-friendly. As previously noted, conventional esterification of starch is usually done under high pH, using harsh chemicals and creating toxic waste, making it expensive and potentially unavailable for large-scale industrial production of modified starches. Unlike conventional modification, dual modification methods, such as PEF–assisted, microwave assisted or enzyme catalyzed esterification, are multifunctional, manageable, less time consuming and have a high reaction efficiency. In addition, these methods avoid the use of organic reagents, which are mild to the environment, as well as the end product. Enzyme catalyzed esterification, however, is much more expensive than the other two methods. Therefore, the dual methods of PEF–assisted or microwave-assisted esterification are potential and promising techniques for starch modification. Nevertheless, the combination of the alternative promising synthesis procedures and its nanostructure, characteristics and novel applications are still far from being exhaustively investigated.

## 5. Conclusions

This review summarizes current determination methods of DS, the merits and shortcomings of diverse modification methods, and the advanced applications for starch esters. From these methods, these emerging dual techniques appear to have the capability to improve DS value, accelerate reaction efficiency, shorten reaction time and explore novel applications. In addition, the dual modification techniques overcome the drawbacks that occur in conventional modification, and can produce all the advantages when modified by the single method, which would gain considerable economic benefits. The conclusion is that dual modification techniques of microwave-assisted and PEF–assisted esterification, which exhibit cost reduction, reagent saving, reaction time shortening, and reaction efficiency promotion, are the two main promising trends for starch modification.

## Figures and Tables

**Table 1:** Effect of conventional and dual modification methods on acetyl content and degree of substitution (DS).

Starch Type	Method	Starch Concentration (*w*/*w*)	Anhydride Content (%)	pH	NaOH (*w*/*w*)	Reaction Time (min)	Acetyl (%)	DS (%)	References
Corn	Conventional	29	8	7.8–8.4	4.0	60–180	2.2–5.3	0.080–0.210	Ayucitra [30]
	Conventional	25	1–3	8.0	3.0	360–1440	-	0.015–0.023	Bhosale, et al. [31]
	Inorganic reagents Catalyzed	30	2–12	8.0–8.4	3.0	~100	3.4–4.7	0.133–0.154	Singh, et al. [13]
	High temperature	32	32	8.0–8.5	3.0	200	-	0.810–2.890	Chi, et al. [32]
Rice	Conventional	31	6	8.0–8.4	3.0	~100	2.3–3.7	0.095–0.144	Sodhi, et al. [10]
	Sodium carbonate assisted	40	1–6 mL	8.0–9.0	-	840	-	0.018–0.045	Bao, et al. [33]
Potato	Conventional	20–40	3	8.0, 8.5	3.0	120–360	-	0.012–0.015	Ruan, et al. [34]
	Sodium carbonate assisted	40	1–6 mL	8.0–9.0	-	840	-	0.017–0.049	Bao, et al. [33]
	Inorganic reagents catalyzed	30	2–12	8.0–8.4	3.0	~100	4.7–6.0	0.180–0.238	Singh, et al. [13]
	PEF–assisted	30–40	6	8.0–8.5	3.0	60	-	0.054–0.130	Hong, et al. [35]
Cassava	Microwave-assisted & High temperature	87–93	3–5	-	No use	3–7	2	0.007–0.051	Jyothi, et al. [36]
	Enzyme catalyzed & Microwave-assisted	50	-	-	No use	2	-	0.330–1.10	Rajan, et al. [37]
Amaranth	Conventional	25	1–3	8.0	3.0	360–1440	-	0.016–0.027	Bhosale, et al. [31]

“-” means that these data have not been listed in the articles. PEF means pulsed electric fields. The volume of 1–6 mL in the second column represent that the anhydride was added 1–6 mL by volume instead of mass percent as in the literature.

**Table 2:** The merits and demerits of diverse determination methods of DS since 2000.

Determination Method	Types of Esters	Merits	Shortcomings	References
Titration	OSA and ACS	Widely used; Better acceptance of its reaction mechanism; Determination of native starch	Complicated process for pretreatment; Increased sample consumption; Time-consuming; Special reaction environment; Color reversion after end point; CO_2_ disturbance; Pyridine used in sometimes	[16,32,38,47]
Back-titration	OSA and ACS	Widely used; Better acceptance of its reaction mechanism; Avoids interference of atmospheric CO_2_ atmosphere; Determination of native starch	Excess alkali; Increased sample consumption; Color reversion after end point;	[10,13,35,48]
Spectrophotometric	ACS	Less sample weight	double color determination; Repeated procedures;	[11,45,49]
FT-IR	ACS	Less sample weight	Determination of series of standard with diverse DS; High cost; Use of titration method; Complicated and fussy procedures	[14]
NMR (involving ^1^H-NMR, ^13^C-NMR)	OSA and ACS	Less sample weight; Simple procedure; Consistent results	the use of internal standards; High cost; Complicated and fussy procedure; Uses of DMSO and chloroform; Determination of DS and DB	[14,41,43,50,51,52]
TGA/IR	ACS	Combination of two instruments	High energy consumption; High temperature; Diverse standard samples	[45]
HS-GC	Cellulose	Less sample weight; Avoids interference of atmospheric CO_2_	-	[46]

OSA, octenyl succinated starch; ACS, acetylated starch; DB, degree of branching; FT-IR, Fourier transform infrared spectroscopy; NMR, nuclear magnetic resonance; TGA/IR, Thermogravimetric analyzer/infrared spectroscopy; HS-GC, Headspace and gas chromatography. “-” means that there’s no use in starch modification in other literature.
